# Sudden cardiac arrest in an athlete due to dilated cardiomyopathy masquerading as athlete's heart: a case report

**DOI:** 10.3389/fcvm.2026.1836946

**Published:** 2026-09-08

**Authors:** Gita Pangestu Hapsari, Brian Mendel, Ira Posangi, Dony Yugo Hermanto

**Affiliations:** 1 Department of Cardiology and Vascular Medicine, National Cardiovascular Center Harapan Kita, Universitas Indonesia, Jakarta, Indonesia; 2 Department of Cardiology and Vascular Medicine, Sam Ratulangi University, Manado, Indonesia; 3 Division of Arrhythmias and Electrophysiology, Department of Cardiology and Vascular Medicine, National Cardiovascular Center Harapan Kita, Universitas Indonesia, Jakarta, Indonesia

**Keywords:** athlete's heart, cardiac magnetic resonance, catheter ablation, dilated cardiomyopathy, implantable cardioverter-defibrillator (ICD), sudden cardiac arrest, torsades de pointes, ventricular arrhythmia

## Abstract

**Background:**

Sudden cardiac arrest (SCA) in athletes is a rare but catastrophic event, most often associated with underlying cardiac pathology. Differentiating physiological remodeling (“athlete's heart”) from pathological conditions such as cardiomyopathy or channelopathy remains a major clinical challenge.

**Case summary:**

A 25-year-old professional athlete presented with recurrent syncope and was found to have frequent premature ventricular complexes with polymorphic ventricular tachycardia and prolonged QT interval. She developed cardiac arrest due to torsades de pointes and was successfully resuscitated. Cardiac magnetic resonance imaging revealed a dilated left ventricle with systolic dysfunction and non-ischemic myocardial fibrosis, consistent with dilated cardiomyopathy rather than physiological adaptation. A 24-hour Holter demonstrated frequent multifocal ventricular ectopy. The patient underwent implantable cardioverter-defibrillator implantation for secondary prevention, followed by successful 3D electroanatomical mapping–guided catheter ablation of premature ventricular complexes originating from the right ventricular outflow tract, resulting in suppression of arrhythmia.

**Conclusion:**

Early identification of pathological substrates in athletes is crucial, as timely intervention, including ICD implantation and catheter ablation can be life-saving.

## Introduction

Sudden cardiac arrest (SCA) in athletes, although rare, is a devastating event most commonly driven by underlying cardiac pathology. Distinguishing physiological remodeling (“athlete's heart”) from occult disease remains a critical challenge, as cardiomyopathies, channelopathies, and myocarditis may be clinically silent and only manifest under exertional stress (1, 2). While hypertrophic cardiomyopathy is the most recognized cause, dilated cardiomyopathy (DCM) and primary electrical disorders are increasingly identified as important substrates. The coexistence of non-ischemic myocardial fibrosis and electrical instability further complicates diagnosis. Ventricular arrhythmias in athletes range from benign to malignant, with high-risk features necessitating prompt evaluation (3, 4). We report a young professional athlete with recurrent syncope and aborted SCA, ultimately diagnosed with non-ischemic DCM and malignant ventricular arrhythmias, underscoring the importance of early recognition and targeted management.

## Case illustration

A 25-year-old woman presented to the emergency department with a second episode of loss of consciousness, which occurred 30 min prior to hospital admission. The patient reported experiencing shortness of breath and palpitations preceding the event. The patient is a professional rollerblade athlete and frequently experienced syncope during periods of intense training. She reported syncope two to three times a year during her athletic career.

**Figure 1:**
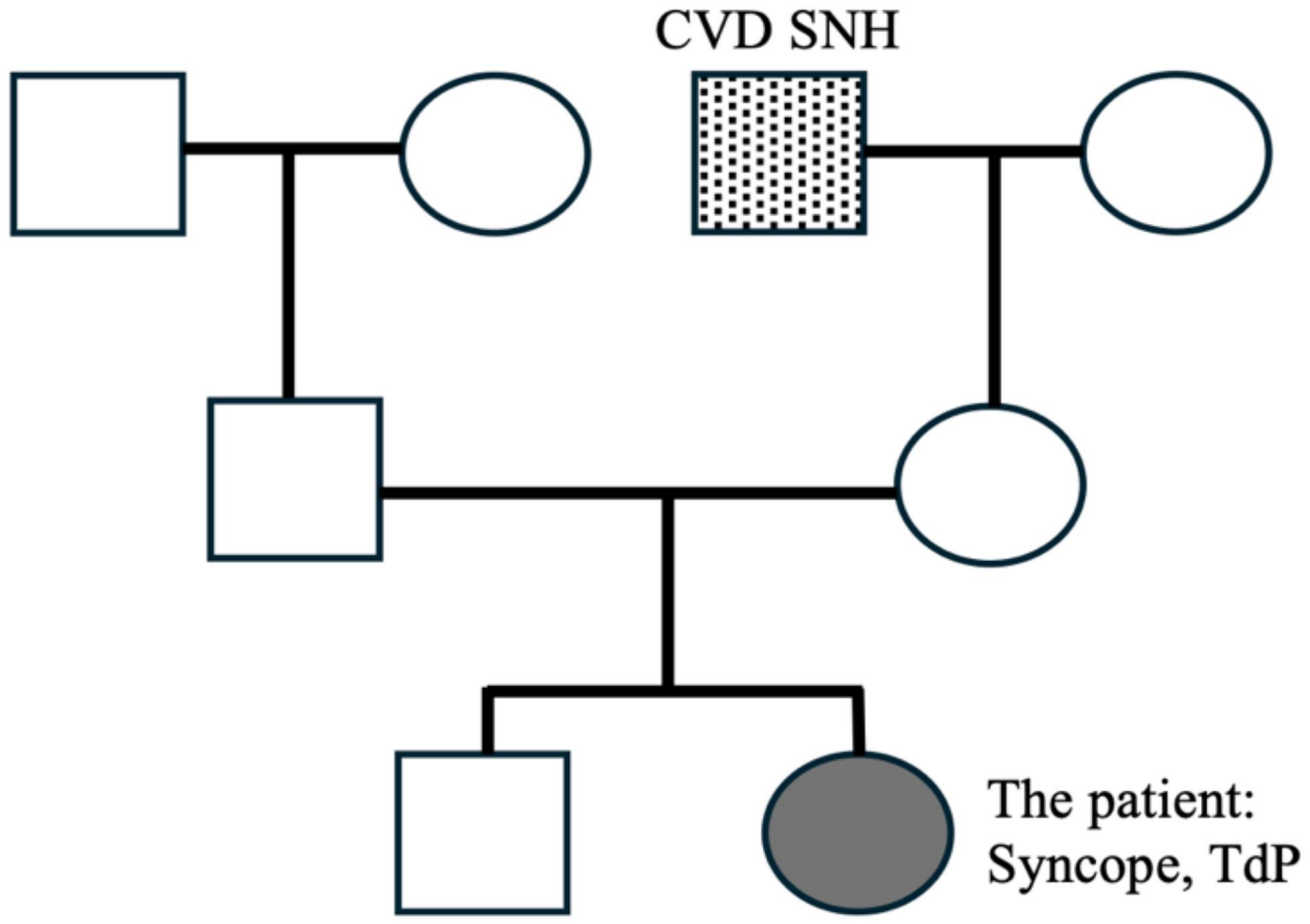
The patient's family pedigree.

She was first diagnosed with arrhythmias six years ago during a routine general check-up for athletes. However, no further treatment was pursued at the time because no episode of syncope occurred. There were no significant past medical conditions, and no family history of sudden death at a young age, other than a grandfather with a family of stroke (Figure 1).

**Figure 2:**
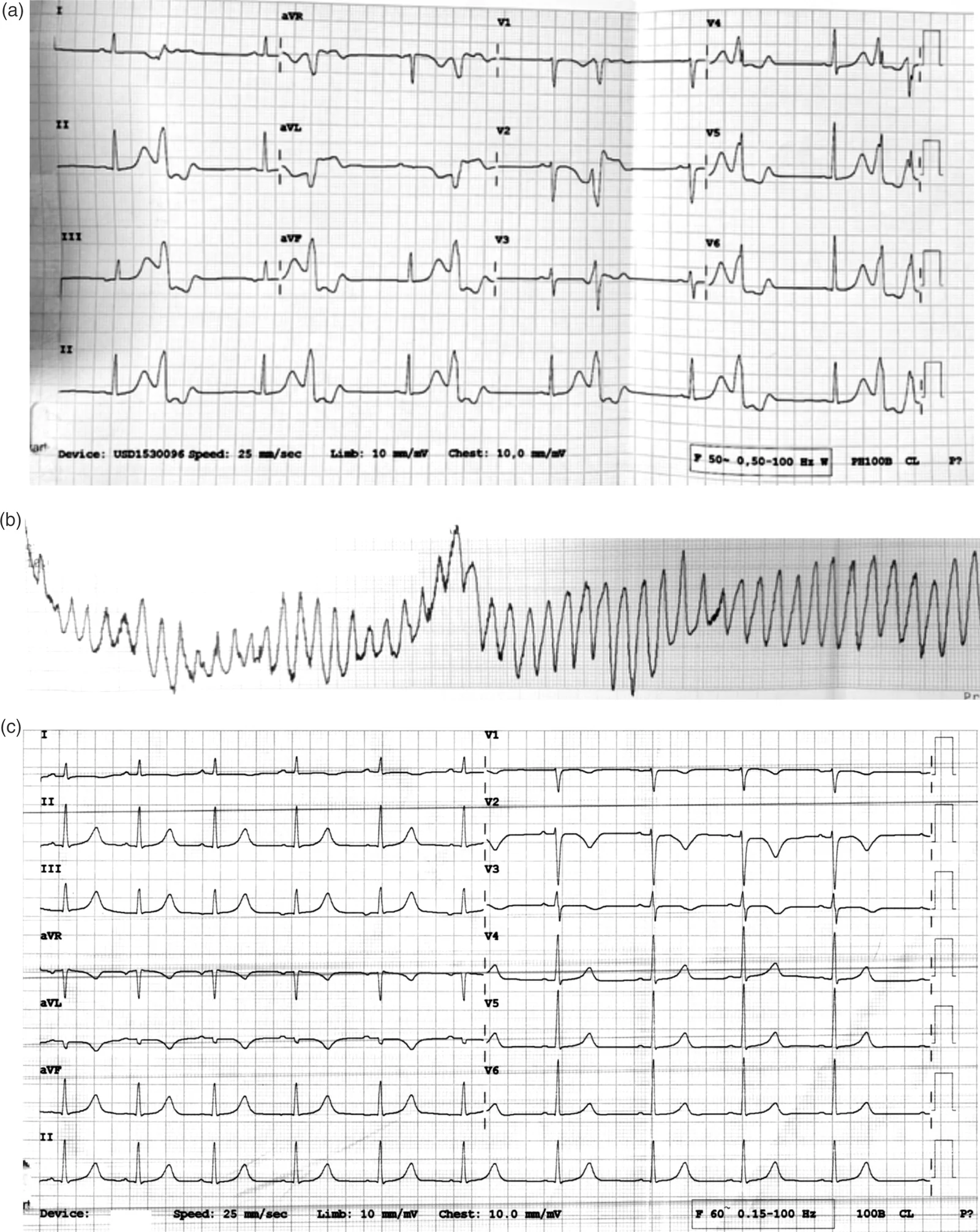
Electrocardiogram in the emergency department. **(a)** Initial ECG showed PVC bigeminy, R-on-T phenomenon with coupling interval of 480 ms (benign) and prolonged QT interval (QTc 584 ms). **(b)** ECG changed into polymorphic VT. **(c)** Electrocardiogram after TdP episode showed SR with prolonged QT interval (QTc 505 ms).

On examination, the patient appeared weak but remained fully conscious. Blood pressure was 113/80 mmHg, with a pulse rate of 87 bpm. A 12-lead electrocardiogram showed SR with PVC bigeminy, R-on-T phenomenon with a coupling interval of 480 ms, and a corrected QT of 584 ms (Figure 2a). During observation in the emergency department, the patient developed cardiac arrest (Figure 2b). Defibrillation was performed and successfully restoring sinus rhythm with persistent QT prolongation and achieving return of spontaneous circulation (Figure 2c).

Laboratory examination showed borderline low potassium 3.8 mmol. The patient was given propranolol 40 mg three times daily and admitted to CVCU. Cardiac Magnetic Resonance Imaging (MRI) showed a dilated left ventricle with normal index volume and severe systolic dysfunction (LVEF 34%) with global hypokinesia. The clinical impression was: (1) dilated cardiomyopathy phenotype with evidence of non-ischemic scar in the mid-to-basal septal region, (2) CMR findings not consistent with athlete's heart, and (3) myocarditis could not be excluded. The final diagnosis was dilated cardiomyopathy (non-ischemic) with systolic dysfunction (CMR LVEF 34%), a history of cardiac arrest survivor with recurrent polymorphic VT and PVC bigeminy originating from the RVOT (R on T).

**Figure 3:**
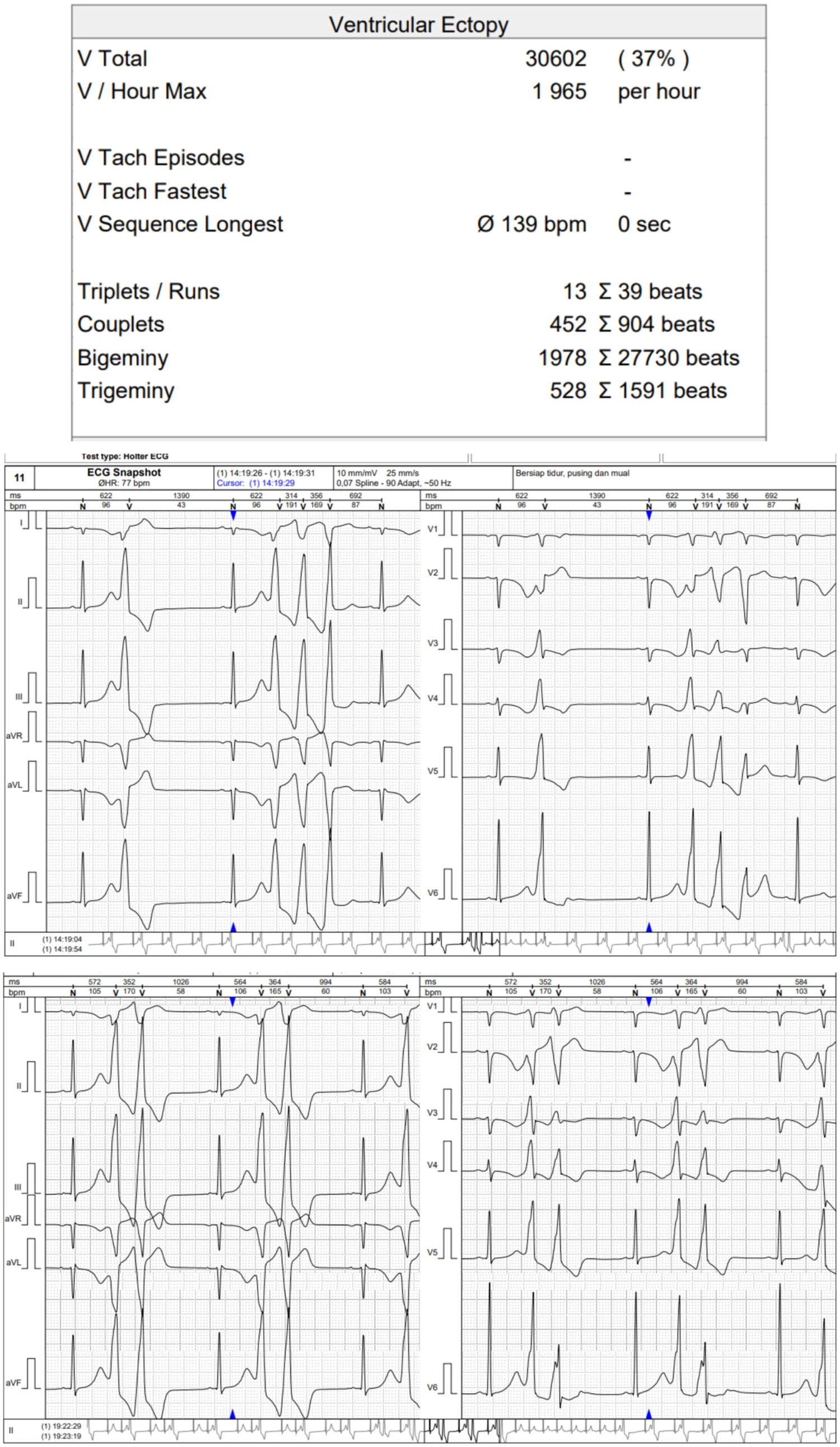
24-hour holter echocardiography showed frequent multifocal benign PVCs originating from the anterior RVOT and the LV summit with PVCs couplet formation, NSVT episodes and prolonged QT interval (QTc 533).

A 24-hour Holter in this patient showed frequent multifocal PVCs with the shortest coupling interval recorded at 476 ms (Figure 3). These ectopic beats originated from the anterior right ventricular outflow tract (RVOT) and the left ventricular summit, and there were episodes of couplet formation, non-sustained ventricular tachycardia (NSVT), and prolonged QT interval (QTc 533 ms). Serial ECG monitoring in the cardiovascular care unit consistently demonstrated sinus rhythm with PVC bigeminy and persistent QT interval prolongation throughout the eight days observation period. Considering the patient's documented episodes of TdP and recurrent syncope, we proceeded with implantation of an implantable cardioverter-defribillator (ICD) for secondary prevention of life-threatening arrhythmias. The device was programmed in VVI mode, with a lower rate of 40 beats per minute and a sensitivity setting of 0.3 mV.

**Figure 4:**
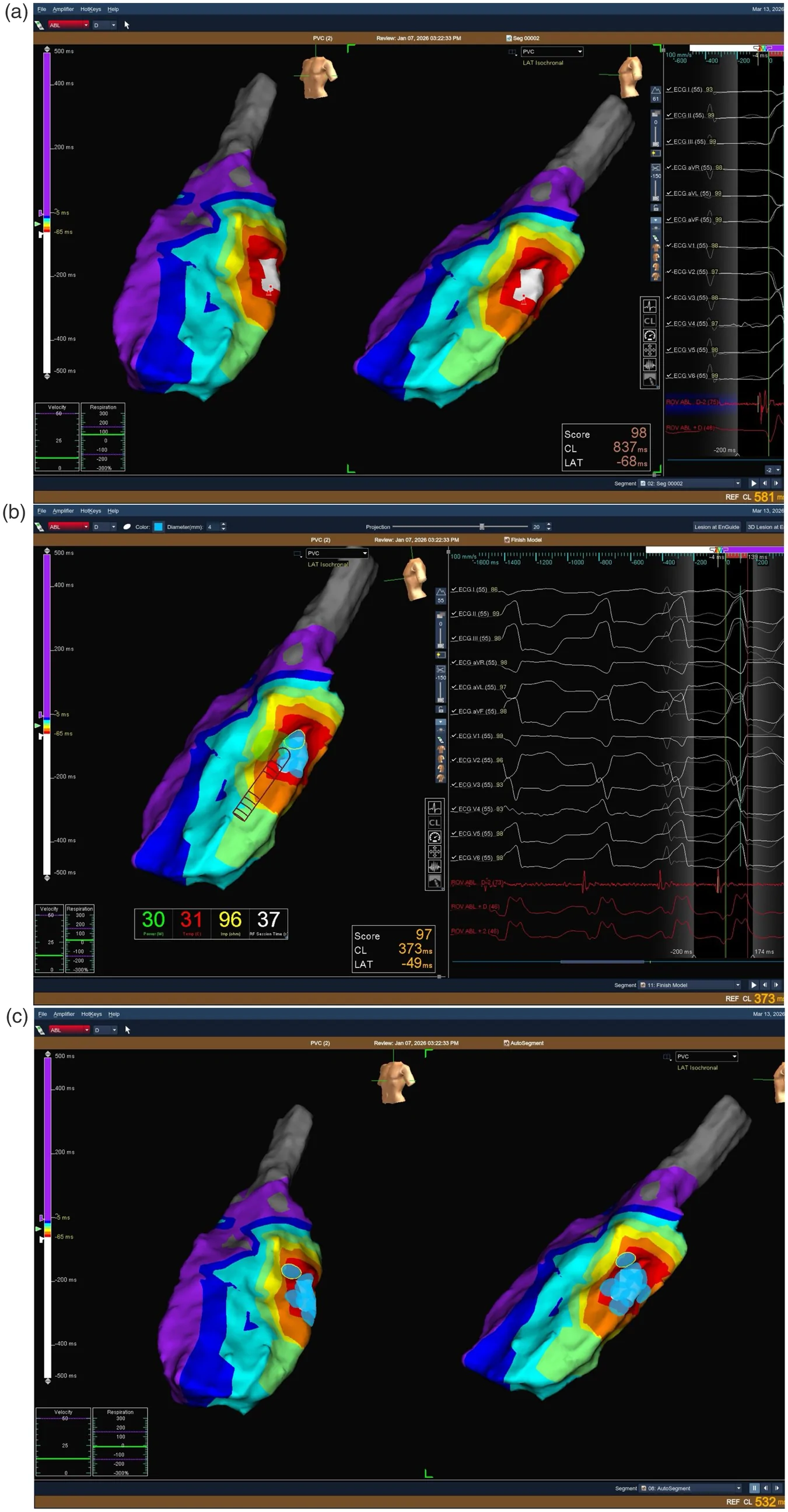
Three-dimensional electroanatomical mapping and ablation of premature ventricular complexes (PVCs) originating from the anteroseptal right ventricular outflow tract (RVOT). **(a)** Local activation time (LAT) mapping using the EnSite 3D electroanatomical system demonstrates the earliest activation at −68 ms in the anteroseptal RVOT, with a unipolar QS pattern and 98% pace-map correlation to the clinical PVC. **(b)** Accelerated ventricular rhythm observed during radiofrequency ablation (30 W, 45 °C, 120 s, irrigation flow rate 17 cc/min), followed by suppression of PVCs. **(c)** Final ablation site corresponding to the earliest activation focus in the anteroseptal RVOT.

Even after ICD placement, the patient was closely monitored. Since serial ECGs showed persistent PVC bigeminy and prolonged QT interval, a 3D ablation was performed (Figure 4). A 12-lead ECG demonstrated sinus rhythm at 70 bpm with frequent premature ventricular complexes (PVCs) characterized by a left bundle branch block morphology, inferior axis, negative deflection in lead I, QS pattern in V1, and transition at V3, suggesting an anteroseptal right ventricular outflow tract (RVOT) origin. The procedure was performed under local anesthesia with sterile preparation of the right inguinal and right jugular regions. Venous access was obtained via two right femoral veins, through which 8F and 6F sheaths were inserted. An 8F FlexAbility irrigated ablation catheter and a quadripolar catheter were advanced to the RVOT and right ventricular apex, respectively. Using the EnSite 3D electroanatomical mapping system, detailed RVOT geometry with voltage and local activation time (LAT) mapping was constructed, revealing the earliest activation at −72 ms in the anteroseptal RVOT with a unipolar QS pattern and 98% pace-map correlation to the clinical PVC. Initial radiofrequency ablation (30 W, 45 °C, 120 s, flow rate 17 cc/min) resulted in transient acceleration followed by suppression of PVCs; however, recurrence was noted after 5 min. Subsequent ablation using similar settings with half-normal saline irrigation (flow rate 10 cc/min) led to further reduction and eventual suppression of PVCs. Comprehensive electrophysiological evaluation demonstrated normal conduction parameters (P–P 997 ms, A–H 135 ms, H–V 42 ms, P–R 178 ms, QRS 101 ms, QT 444 ms), retrograde block during ventricular pacing, RV effective refractory period of 290 ms, and normal atrioventricular nodal and atrial refractory properties. Sinus node recovery times were within normal limits. After a 20-minute observation period without PVC recurrence, the procedure was concluded. Total dose-area product was 12.1 Gy·cm^2^ with a fluoroscopy time of 32 min and 23 s. Overall, findings were consistent with frequent PVCs originating from the anteroseptal RVOT, successfully treated with 3D-guided catheter ablation, with preserved sinus and atrioventricular nodal function. At the 3-month follow-up, the patient remained asymptomatic and had resumed light gym-based exercise without limitations. Transthoracic echocardiography demonstrated improvement in left ventricular systolic function, with an LVEF of 48%, TAPSE of 25 mm, LVIDd of 50.9 mm, and LVIDs of 36.6 mm.

## Discussions

Maron et al. (1) and Chandra et al. (5) identified the causes of non-traumatic sudden death among athletes, with 80% of cases attributed to cardiac causes and the remainder to non-cardiac causes. In young athletes (<35 years), the most common cause is hypertrophic cardiomyopathy (HCM) at 33%, followed by congenital coronary anomalies, channelopathies and dilated cardiomyopathy (1, 6, 7). The patient belongs to young athlete groups who experienced cardiac arrest due to TdP in the context of QTc prolongation and non-ischemic left ventricular dysfunction, most likely falling within the “others” group in the sudden cardiac death etiology specifically channelopathy and dilated cardiomyopathy. These two conditions are now recognized as important causes of SCD in athletes according to the ESC/EAPC 2020 guidelines (3). The findings of left ventricular dysfunction LVEF 34% and QT prolongation (QTc 584 ms) in this patient illustrate an arrhythmogenic substrate of both structural and electrical origin, both of which are highly relevant to sudden cardiac death in athletes.

Approximately 10% of athletes demonstrate PVC during pre-participation cardiovascular screening. Athletes involved in high-intensity endurance sports face a fivefold increased risk of developing atrial fibrillation. However, overall prevalence of ventricular arrhythmias is not significantly different between athletes and sedentary individuals (8, 9). D’Ambrossio et al. (2024) (10) delineated how exercise-induced sympathetic activation increases the likelihood of abnormal electrical activity, especially in the presence of myocardial scarring or structural heart disease. Neurohormonal shifts of exertion amplify the risk of malignant arrhythmias and sudden cardiac death, as evidenced by patient's progression to life-threatening ventricular tachycardia during physical activity.

The differences in cardiac structural integrity from cardiac MRI and arrhythmic vulnerability among three distinct categories: normal myocardium, physiologically remodelled “athlete's heart,” and heart afflicted by structural or electrical pathology. Arrhythmogenic epicardial scarring often patchy and non-ischemic can serve as a critical substrate for potentially fatal ventricular arrhythmias in athletes. These structural abnormalities may develop because of extreme cardiac remodeling from sustained high-intensity exercise, producing areas of fibrosis particularly in the epicardial RVOT or inferolateral left ventricle (9, 11).

**Figure 5:**
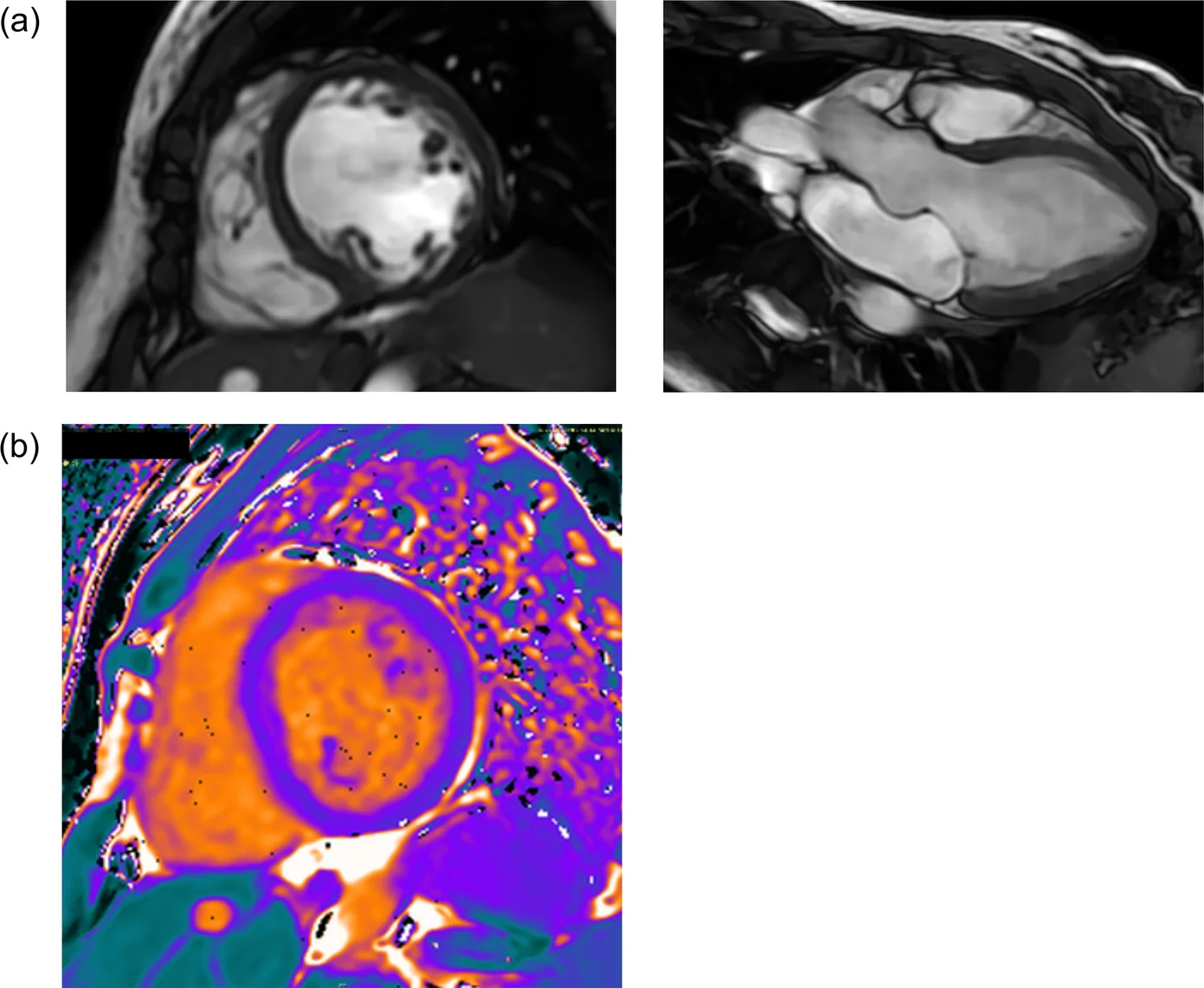
Cardiac MRI findings. **(a)** Intramyocardial fibrosis. **(b)** Increased T1 relaxation time (basal septal 1,112 ms).

In this case, cardiac MRI findings fulfill the criteria for non-ischemic fibrosis based on Late Gadolinium Enhancement (LGE), characterized by intramyocardial fibrosis not located within the typical epicardial regions affected by ischemic heart disease. This intramyocardial pattern of fibrosis may be associated with chamber dilation, as the LV showed increased dimensions (62–64 mm) (Figure 5a). These findings could still be consistent with a dilated cardiomyopathy phenotype, however concomitant increased T1 relaxation time (basal septal 1,112 ms) suggests underlying microinflammation, increasing the suspicion of myocarditis (Figure 5b). The cardiac MRI findings did not meet the criteria for physiological remodeling typical of an athlete's heart (3–6).

Stratification of VAs in athletes relies heavily on ECG morphology. Monomorphic arrhythmias, particularly those with a LBBB morphology inferior axis or a RBBB configuration with narrow QRS complex are most frequently observed in athletes and are generally considered benign. Polymorphic VAs with a non-LBBB inferior axis are rare in this population and are indication of an increased risk for underlying structural or electrical disease (7, 10). This case initially demonstrated LBBB in inferior axis and polymorphic ventricular premature beats, however progression to TdP represented an uncommon and high-risk arrhythmic pattern. Red flag features nonstandard or atypical arrhythmia morphologies, multifocal or consecutive PVC, short coupling intervals, and the persistence or worsening of arrhythmias during exercise testing (3, 4).

The 2020 ESC Guidelines on Sports Cardiology and Exercise in Patients with Cardiovascular Disease stated that if advanced investigations confirm a condition associated with increased risk for sudden cardiac death, the guidelines indicate restricting competitive sports and focusing on medical therapy and non-competitive exercise (3).

Catheter ablation has emerged as an increasingly utilized strategy for the management of ventricular arrhythmias (VAs) over the past several decades. Procedural success is most commonly defined by the achievement of non-inducibility, which has been reported in approximately 38% to 74% of cases (12). Despite this, recurrence of ventricular tachycardia (VT) remains substantial, reaching up to 58% in patients with non-ischemic cardiomyopathy. Nevertheless, catheter ablation has demonstrated important clinical benefits, including reduction in antiarrhythmic drug dependence, decreased implantable cardioverter-defibrillator (ICD) shock burden, and effectiveness in terminating electrical storm (4, 13). Notably, many studies evaluating ablation outcomes in non-ischemic cardiomyopathy have included heterogeneous populations, particularly those encompassing various forms of dilated cardiomyopathy, which may contribute to the observed variability in outcomes when compared with VT ablation in ischemic cardiomyopathy (12, 14, 15). Premature ventricular contraction (PVC)-induced cardiomyopathy was considered as an important differential diagnosis given the patient's high PVC burden (37%). However, the presence of non-ischemic mid-myocardial fibrosis in the basal-to-mid interventricular septum on cardiac magnetic resonance imaging, together with a reduced left ventricular ejection fraction (34%), is more consistent with an underlying structural myocardial disease rather than isolated PVC-induced cardiomyopathy. Furthermore, ICD therapy remains indicated even if left ventricular function improves following optimal medical therapy or catheter ablation, as the patient had already experienced sudden cardiac arrest, torsades de pointes, and polymorphic ventricular tachycardia. In accordance with the 2022 ESC Guidelines (4), survivors of cardiac arrest due to ventricular tachyarrhythmias without a reversible cause have a Class I indication for ICD implantation for secondary prevention, irrespective of subsequent recovery in left ventricular ejection fraction.

In young patients undergoing conventional transvenous ICD implantation, the cumulative lifetime risk of lead failure, infection, and repeated lead revisions is substantial, making lead-sparing technologies an attractive alternative. The subcutaneous ICD (S-ICD) eliminates the need for transvenous leads and has demonstrated comparable efficacy for ventricular arrhythmia termination while significantly reducing lead-related complications. However, its inability to provide anti-tachycardia pacing (ATP), bradycardia pacing, or cardiac resynchronization therapy limits its use in patients with pacing requirements. More recently, the extravascular ICD (EV-ICD), with a substernal lead position, has emerged as a promising option that combines the advantages of avoiding transvenous leads with the capability to deliver ATP and backup pacing (16). In the EV-ICD Pivotal Study (17), the device achieved 100% successful defibrillation testing, ATP successfully terminated 77.1% of ventricular tachycardia episodes, and freedom from major system- or procedure-related complications was 89% at 3 years, although longer-term experience remains limited. Therefore, in carefully selected young patients without indications for cardiac resynchronization therapy, S-ICD and EV-ICD represent valuable alternatives to conventional transvenous ICDs, with device selection guided by the anticipated need for pacing, arrhythmia characteristics, and long-term lead management.

## Conclusions

Non-ischemic dilated cardiomyopathy may initially present with malignant ventricular arrhythmias and sudden cardiac arrest, underscoring the importance of early recognition and comprehensive evaluation. Optimal diagnosis and risk stratification require a multidisciplinary approach involving cardiologists, cardiac electrophysiologists, cardiovascular imaging specialists, radiologists, genetic specialists when indicated, and sports medicine physicians to identify the underlying etiology, assess arrhythmic risk, and guide recommendations regarding physical activity. Timely implementation of evidence-based therapies, including implantable cardioverter-defibrillator (ICD) implantation and catheter ablation when appropriate, together with coordinated long-term follow-up, is essential to prevent recurrent life-threatening events and improve clinical outcomes.

## Patient’s perspectives

The patient was able to resume gym-based exercise without any symptoms and reported a high level of satisfaction with the outcome of the three-dimensional catheter ablation procedure.

## Data Availability

The original contributions presented in the study are included in the article/Supplementary Material, further inquiries can be directed to the corresponding author.
